# Detailed Analysis of Sequence Changes Occurring during *vlsE* Antigenic Variation in the Mouse Model of *Borrelia burgdorferi* Infection

**DOI:** 10.1371/journal.ppat.1000293

**Published:** 2009-02-13

**Authors:** Loïc Coutte, Douglas J. Botkin, Lihui Gao, Steven J. Norris

**Affiliations:** 1 Department of Pathology and Laboratory Medicine, University of Texas Medical School at Houston, Houston, Texas, United States of America; 2 Department of Microbiology and Molecular Genetics, University of Texas Medical School at Houston, Houston, Texas, United States of America; Medical College of Wisconsin, United States of America

## Abstract

Lyme disease *Borrelia* can infect humans and animals for months to years, despite the presence of an active host immune response. The *vls* antigenic variation system, which expresses the surface-exposed lipoprotein VlsE, plays a major role in *B. burgdorferi* immune evasion. Gene conversion between *vls* silent cassettes and the *vlsE* expression site occurs at high frequency during mammalian infection, resulting in sequence variation in the VlsE product. In this study, we examined *vlsE* sequence variation in *B. burgdorferi* B31 during mouse infection by analyzing 1,399 clones isolated from bladder, heart, joint, ear, and skin tissues of mice infected for 4 to 365 days. The median number of codon changes increased progressively in C3H/HeN mice from 4 to 28 days post infection, and no clones retained the parental *vlsE* sequence at 28 days. In contrast, the decrease in the number of clones with the parental *vlsE* sequence and the increase in the number of sequence changes occurred more gradually in severe combined immunodeficiency (SCID) mice. Clones containing a stop codon were isolated, indicating that continuous expression of full-length VlsE is not required for survival *in vivo*; also, these clones continued to undergo *vlsE* recombination. Analysis of clones with apparent single recombination events indicated that recombinations into *vlsE* are nonselective with regard to the silent cassette utilized, as well as the length and location of the recombination event. Sequence changes as small as one base pair were common. Fifteen percent of recovered *vlsE* variants contained “template-independent” sequence changes, which clustered in the variable regions of *vlsE*. We hypothesize that the increased frequency and complexity of *vlsE* sequence changes observed in clones recovered from immunocompetent mice (as compared with SCID mice) is due to rapid clearance of relatively invariant clones by variable region-specific anti-VlsE antibody responses.

## Author Summary

Lyme borreliosis is the most common vector-transmitted infection in Europe and North America, and is caused by the spirochete *Borrelia burgdorferi* and other closely related *Borrelia* species. Lyme disease *Borrelia* have an elaborate mechanism for varying the sequence of VlsE, a surface-localized, immunogenic lipoprotein. This antigenic variation is thought to be important in immune evasion and thus in the ability of Lyme disease *Borrelia* to cause long-term infection. In this study, we examined 1,399 *B. burgdorferi* clones isolated from infected immunocompetent and immunodeficient mice to gain a better understanding of the rate and variety of VlsE sequence changes that occur during infection. We determined that clones with few or no VlsE sequence changes are rapidly cleared in mice with active immune responses, whereas clones with many VlsE changes persist. The *vls* antigenic variation system can utilize any of the 15 silent cassette sequences as sequence “donors,” and does not exhibit obvious preferences in the location of changes within the *vlsE* cassette region or the types of VlsE sequence variations found in different tissues, such as in joints or in the heart. Our findings provide further evidence that the *vls* locus represents a remarkably robust recombination system and immune evasion mechanism.

## Introduction

Lyme borreliosis is caused by *Borrelia burgdorferi* and other members of the genus *Borrelia*, and is the most prevalent vector-borne disease in the United States [1]. Spirochetes are transmitted to mammalian hosts by *Ixodes* ticks, causing a local skin infection, usually accompanied by a lesion called erythema migrans. As the infection advances, *Borrelia* disseminate into deeper tissues despite a strong immune response against the pathogen [2]–[6]. However, Lyme disease *Borrelia* are able to escape clearance and cause disease manifestations (including neurologic, arthritic, cardiovascular, and dermatologic symptoms) for months to years after the initial infection.

Antigenic variation results from changes in surface antigen genes that occur during the course of infection at rates higher than the expected mutation frequency [7]. This mechanism is particularly important for organisms that cause long-term or repeated infections. Pathogens with antigenic variation systems are able to evade the immune response, thus gaining a selective advantage over their more antigenically stable counterparts and posing a challenge in the development of vaccines. Influenza virus [8] , HIV [9], *Neisseria gonorrhoeae* and *N. meningitidis* [10], *Mycoplasma synoviae* [11], *Mycoplasma pulmonis* [12], *Anaplasma marginale* [13], *Borrelia burgdorferi* [14], *Borrelia hermsii* [15],[16], *Treponema pallidum* [17], *Campylobacter jejuni* [18], *Candida* species [19], *Plasmodium falciparum* [20] and *Trypanosoma brucei* [21] are some examples of viruses, bacteria, fungi and parasites that avoid immune clearance through antigenic variation.

A surface-exposed lipoprotein, VlsE, contributes to immune evasion and persistence of Lyme borreliosis organisms in infected mammalian hosts through an elaborate antigenic variation mechanism [14], [22]–[25] . The Vmp-like sequence (*vls*) locus of *B. burgdorferi* B31 is located on the linear plasmid lp28-1. The *vls* locus is composed of an expression site (*vlsE*) encoding the 35 kDa lipoprotein VlsE and a contiguous array of 15 unexpressed (silent) *vls* cassettes. The silent cassettes have high homology to the central cassette region of *vlsE* (90.0% to 96.1% nucleotide sequence identity and 76.9% to 91.4% predicted amino acid sequence identity), and most of the sequence differences are concentrated in six variable regions within each cassette [22]. Clones lacking lp28-1 exhibit an intermediate infectivity phenotype, characterized by decreased persistence and aberrant tissue distribution in immunocompetent mice but no change in virulence in SCID mice [24],[26],[27]. Recent studies by Bankhead and Chaconas [23] demonstrated that removal of the *vls* locus by telomere-mediated truncation resulted in the same phenotype as the loss of lp28-1, whereas truncation of the other end of the plasmid had no detectable effect on mouse infection by needle inoculation. These results support the role of the *vls* locus in immune evasion.

Previous analysis of a limited number of clones recovered from experimentally infected mice or rabbits indicated that *vlsE* sequence variation occurs within 4 days and continues throughout the course of infection [25],[28]. Only the cassette region of the *vlsE* is subject to sequence variation during these recombination events. Segments of the *vls* silent cassette sequences replace portions of the *vlsE* cassette region through a gene conversion process, such that the sequence and organization of the silent *vls* cassettes remain unaltered [14]. *vlsE* antigenic variation has not been detected during *in vitro* culture or during tick infection, but occurs during mammalian infection in both immunocompetent and severe combined immunodeficiency disease (SCID) animals [14],[22],[25],[29],[30]. Attempts to induce *vlsE* recombination *ex vivo* have been unsuccessful. Therefore, the induction of *vlsE* recombination occurs through an as yet unidentified signaling mechanism.

Most sequence changes that occur during *vlsE* recombination events are localized within the six variable regions. The six invariable regions within the cassette region [22] contain relatively few variable codons and are likely to be important in preserving overall protein structure and biological function [31]. The variable regions form random coil structures on the membrane distal surface of the protein where antibody interactions are most likely [31]. Immunoglobulins specific for these regions are generated during the course of infection [32]. Also, the resulting variants exhibit decreased reactivity to antisera raised against a recombinant form of the *vlsE* cassette region from the parental clone; indicating that the sequence changes result in real antigenic variation [22]. The mechanisms that promote the selectivity and unidirectionality of gene conversion in the *vls* locus have not been identified.

In the current study, *B. burgdorferi* clones acquired 4 to 365 days following infection of immunocompetent or SCID mice were examined to gain a better understanding of the *vlsE* recombination process. The results provide further evidence of the remarkable randomness of recombination events occurring within the *vlsE* cassette region.

## Results/Discussion

### Prolonged persistence of clones possessing the parental *vlsE* sequence in SCID mice

We analyzed the *vlsE* cassette region sequences of 1399 clones recovered during the time course of infection of immunocompetent C3H/HeN and immunocompromised C3H/HeN SCID and CB-17 SCID mice (Table 1). These results comprised 85 previously reported clones [14],[22] and 1320 clones derived during this study. The earlier studies were performed with *B. burgdorferi* B31clone 5A3 and utilized CB-17 SCID mice, whereas our recent analyses used clone B31 5A4 and C3H/HeN SCID mice. Although clone 5A3 is lacking plasmids lp28-2 and lp56 and CB-17 mice have a different genetic background than C3H/HeN, the results obtained were comparable (data not shown); therefore, the results obtained with the two *B. burgdorferi* B31 clones and the two SCID mouse strains were combined to increase the number of isolates and time points analyzed without necessitating additional animal experiments. Bladder, heart, joint, ear and back skin biopsy isolates were obtained to examine the rate and nature of *vlsE* recombination occurring in different tissues. In the current analysis, we focused on days 4, 7, 10, 14 and 28 post-inoculation because individual recombination events can be discerned more commonly at these earlier time points.

**Table 1:** Number of *vlsE* sequences analyzed from different tissues and time points during experimental infection of immunocompetent C3H/HeN mice or SCID mice with *B. burgdorferi* B31.

Days post infection	No. of sequences									
	C3H/HeN					SCID^a^				
	Bladder	Heart	Joint	Ear	Skin	Bladder	Heart	Joint	Ear	Skin
4	NC^b^	NC	NC	NC	43*	NC	NC	NC	NC	23^§^*
7	72	71	76	NC	6*	4	15	20	NC	ND
10	61	59	60	60	ND^c^	34	27	28	24	ND
14	49	40	57	55	10*	21	39	31	47	9^§^*
21	ND	ND	ND	ND	48*	ND	ND	ND	ND	ND
28	42*	49*	40*	19*	17*	44^§^*	41^§^*	20^§^*	ND	23 ^§^*
214	ND	ND	ND	ND	7*	ND	ND	ND	ND	ND
365	3*	ND	ND	ND	5*	ND	ND	ND	ND	ND

*B. burgdorferi* B31 Clones 5A4 or 5A3 (asterisks) were injected subcutaneously 10^5^/mouse at the base of the tail. Groups of 4 to 6 mice were sacrificed on the indicated days post infection.

Cultures from the tissue sites (urinary bladder, heart, tibiotarsal joint, ear pinnae and skin punch biopsies) were acquired under aseptic conditions and clones obtained by subsurface colony formation in agar plates.

a SCID mice were either C3H/HeN SCID or CB-17 SCID (^§^).

b NC = no positive cultures obtained.

c ND = cultures not done.

**Figure 1:**
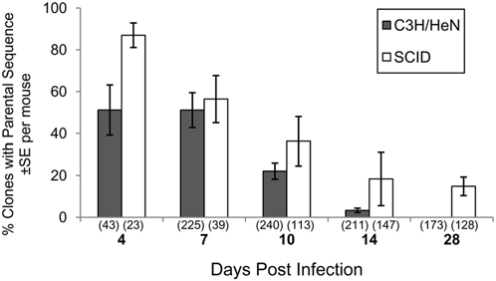
*B. burgdorferi* clones having the parental *vlsE* sequence are cleared more rapidly during infection of immunocompetent C3H/HeN mice than in immunodeficient SCID mice. The numbers in parentheses represent the total number of clones at each time point.

As previously observed [25], we found that clones had already undergone *vlsE* recombination within 4 days post infection in both immunocompetent and SCID mouse models (Figure 1). In immunocompetent mice, only 50% of the retrieved spirochetes retained the parental *vlsE* sequence after 4 days of infection, meaning that the remaining 50% of the population had already incurred one or more *vlsE* recombination events. By 14 days post infection, clones with the parental *vlsE* sequence were few in number (3% of all examined) and were not detected at 28 days after inoculation (Figure 1). In SCID mice, 87% of the recovered bacteria retained the parental *vlsE* sequence at 4 days post infection. The proportion of parental bacteria decreased more slowly than in immunocompetent mice, such that parental clones represented 18.7% and 15% of the populations recovered in SCID mice at 14 and 28 days post infection, respectively.

The parental bacteria thus persisted longer in the absence of an adaptive immune response. The rapid clearance of the parental genotype in immunocompetent mice actually preceded the detection of anti-VlsE antibodies by ELISA 8 days post infection in C3H/HeN mice [33]; this result suggests that the anti-variable region immune responses are present in small quantities within a few days of infection and are extremely effective in eliminating clones expressing the corresponding variable region epitopes. The more gradual decrease in the proportion of parental clones in SCID mice most likely represents the simple dilution of the initial genotype by variant clones.

### Reduced persistence of clones showing the parental *vlsE* sequence in heart and bladder in immunocompetent mice

**Figure 2:**
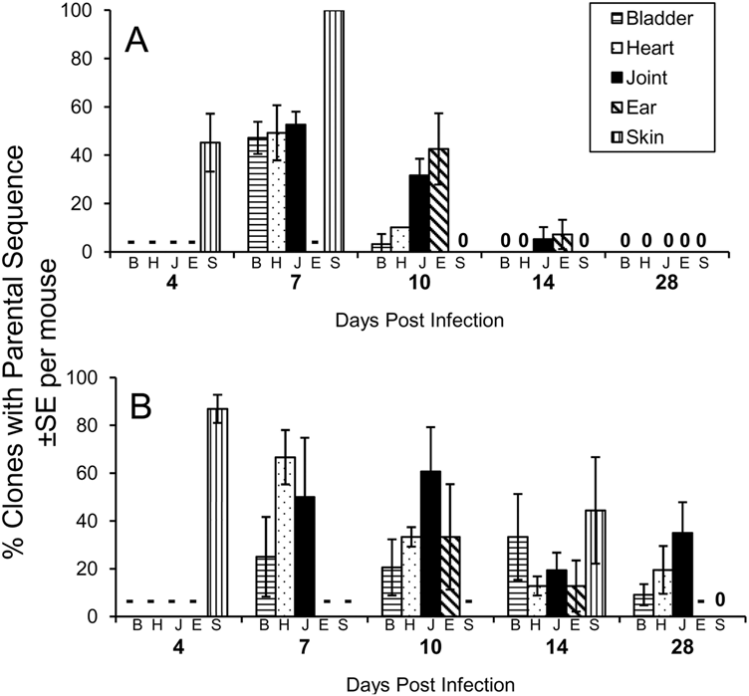
Relative persistence of clones retaining the parental *vlsE* sequence at different tissue sites during the course of infection of C3H/HeN and SCID mice. Panel (A) represents data obtained from C3H/HeN mice, whereas Panel (B) contains data from SCID mice. Results for each time point are presented in the order shown (bladder, heart, joint, ear, and skin). - No data available (no positive culture obtained, or culture not done). 0 No parental sequences identified for that tissue and timepoint.

At 4 days post infection, only the back skin biopsies (taken at a site distant from the inoculation site) and blood samples (not shown) exhibited positive culture results in C3H/HeN and SCID mice, suggesting that the spirochete had not colonized the other tissues examined to a detectable extent at this early time point (Table 1, Figure 2). At 7 days post infection in both mouse models, samples from the ear pinnae did not yield positive cultures, while all other sites were culture positive; this result indicates that the colonization of the external ear takes more time than the other tissues tested (Table 1, Figure 2). In comparing the different tissues, the proportion of clones with the parental *vlsE* sequence was not significantly different in either mouse model at day 7 post infection. Thereafter in C3H/HeN mice (but not in SCID mice), the proportion of parental clones dropped drastically in bladder, heart, and skin samples between 7 to 10 days post infection and in joint and ear samples between 10 and 14 days post infection (Figure 2A). These results indicate that the parental bacteria are cleared more quickly (or, alternatively, undergo more rapid *vlsE* recombination) in bladder, heart and skin than in joint and ear tissues in immunocompetent mice.

The more rapid clearance of *B. burgdorferi* with the parental *vlsE* sequence in heart, bladder and skin may indicate a higher accessibility of the bacteria to the adaptive immune system in these sites. Alternatively, bacteria in joint and ear tissues may localize in immunoprotective niches (*e.g.* in relatively avascular or highly collagenous regions) that allow those expressing the parental *vlsE* sequence to survive longer. In previous studies, it has been demonstrated that, in immunocompetent mice, *B. burgdorferi* clones lacking lp28-1 [24],[26],[33] or the *vls* locus [23] persist for longer periods in joint tissue than in other tissues. In contrast, organisms with these genotypes are able to infect and disseminate to all tested tissues in SCID mice. These results support the concept that immune evasion mechanisms provided by VlsE expression and sequence variation promote the survival of *B. burgdorferi*, but that bacteria that either do not express VlsE or have not undergone sequence variation are relatively protected in some tissues, such as those present in the tibiotarsal joint.

### Accelerated accumulation of *vlsE* sequence changes in the presence of the adaptive immune response

**Figure 3:**
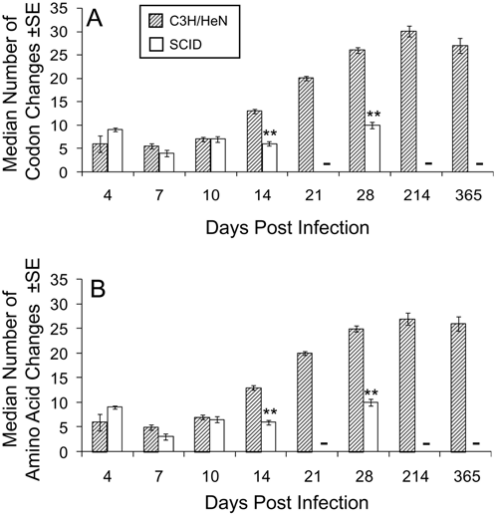
Median number of *vlsE* codon changes and predicted amino acid changes in variants during the time course of infection of C3H/HeN or SCID mice. Clones with the parental *vlsE* sequence were excluded from this analysis. (A) *vlsE* codon changes. (B) Predicted amino acid changes. ** indicates a significant difference (P<0.01) between organisms from C3H/HeN and SCID mice at the time points indicated, as calculated by unpaired Student's t test. - No data available (culture not done).

We analyzed more specifically the population of variants (n = 1,073) by excluding all clones with the parental *vlsE* sequence (n = 326). The group of variants included 921 ‘unique’ variant sequences and 158 additional sibling clones (i.e. variants with the same sequence in the same tissue specimen). The number of codon changes observed was paralleled closely by the number of amino acid changes (Figure 3), in concordance with the high proportion of nonsynonymous codon differences in the silent cassettes that serve as templates for these sequence changes.

In immunocompetent mice, the median number of codon or amino acid changes in the *vlsE* variant clones did not increase significantly between 4 to 10 days post infection, but at 14, 21 and 28 days post infection the number of changes increase rapidly and significantly (Figure 3A; P<0.001 for differences in the median number of changes on days 10 and 14, days 14 and 21, and days 21 and 28). There was no significant difference in the median number of changes at 28 days and 365 days post infection. The process of recombination in *vlsE* is still functional after 28 days, but the number of changes relative to the parental strains becomes asymptotic [25], as addressed further below. In immunocompromised mice, the number of codon or amino acid changes in *vlsE* was not significantly different when comparing 4 days and 14 days post infection (P>0.05). On days 14 and day 28, the number of changes was significantly lower in SCID mice than in immunocompetent C3H/HeN mice (P<0.001, Figure 3A and B). Thus, the immune pressure provided by the adaptive immune system not only results in the more rapid elimination of parental clones (Figure 1), but also selects for clones with more sequence changes and hence antigenic differences. These findings are again consistent with the observation that the presence of lp28-1 or, more specifically, an intact, functional *vls* locus [23] is required for long-term survival of *B. burgdorferi* in immunocompetent mice, but not in SCID mice.

### Analysis of recombination events

Each of the 1,073 clones that had undergone *vlsE* sequence variation was examined individually to provide a global view of the length, location, and most likely silent cassette sources of the recombination events. As in previous analyses of *vlsE* sequence variation, segmental gene conversion events were observed; in no instance was the entire cassette region of *vlsE* replaced by a silent cassette. In most cases, the sequence changes could be attributed to a particular silent cassette sequence or set of potential donor sequences. However, in many instances, the donor sequence could not be identified unequivocally due to the high degree of sequence redundancy among the silent cassettes. Tentative identifications of recombination events and the corresponding donor sequences were thus based on those sequence alignments that incorporated the longest stretch of sequence changes (minimal recombination event) flanked by regions that were shared between the parental *vlsE* and *vls* silent cassette sequences (constituting the maximal possible recombination event).

**Figure 4:**
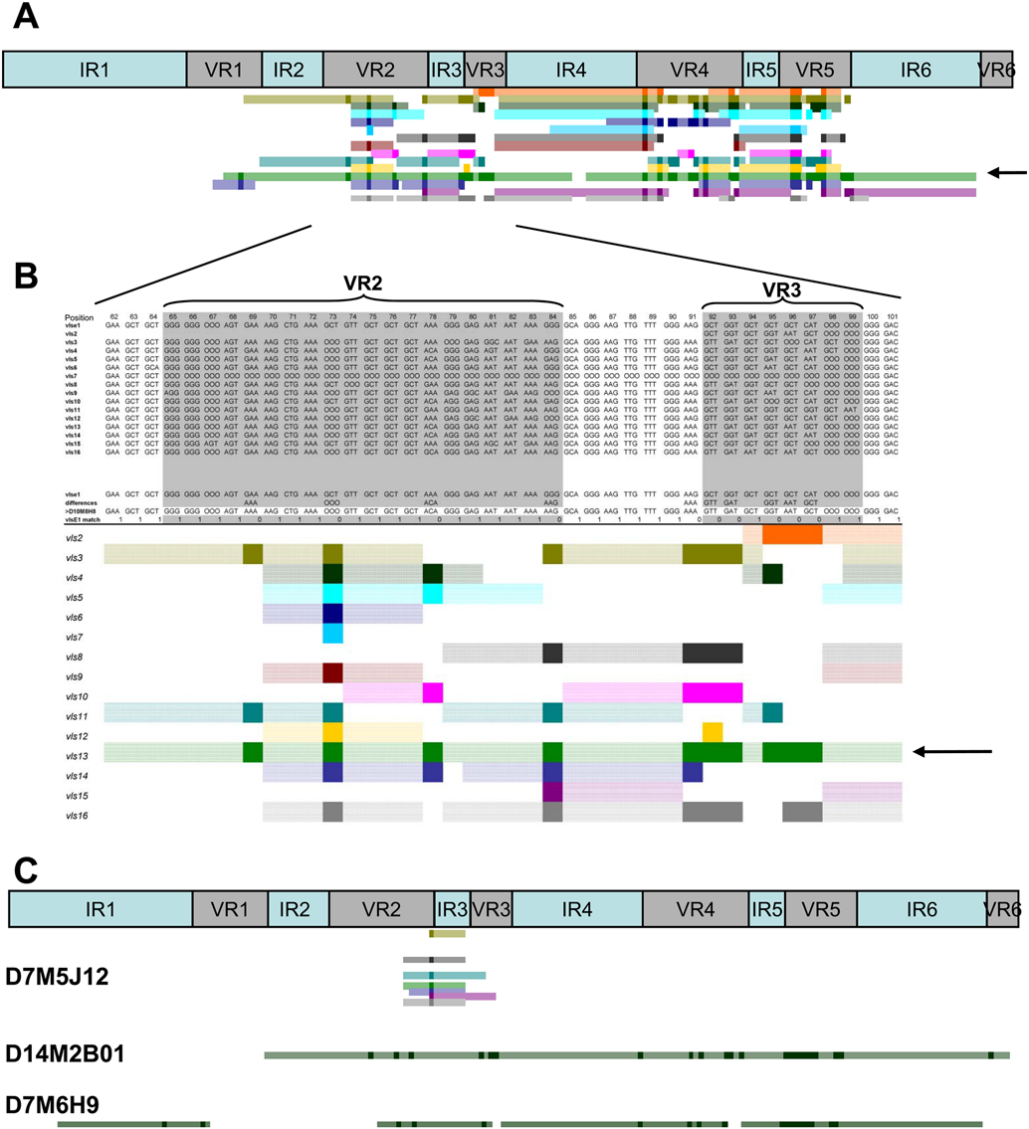
Schematic representation of *vlsE* recombination patterns. (A) Upper panel represents the locations of the 6 invariable regions (IR) and the 6 variable regions (VR) within the *vlsE* cassette region. Lower panel illustrates the pattern of recombination of clone D10M8H8 showing sequence changes between VR1 to VR5. (B) Magnified region of D10M8H8 recombination pattern. The top portion of the diagram shows the alignment between the parental *vlsE* sequence (*vlsE1*), the *vls* silent cassettes *vls2* through *vls16*, and the *vlsE* variant. The line “differences” highlights the difference between the *vlsE* and the variant sequences. In the lower panel, each colored line schematically represents regions of the 15 silent cassette sequences which could be involved in the variant sequence changes. Each dark colored block represents a region of sequence change within the variant sequence that is present in the selected silent cassette sequence. The light colored regions in each line represent segments adjacent to sequence changes that are identical in *vlsE1*, the variant and the selected silent cassette sequence (i.e. the maximal possible recombination region). From this analysis, two likely recombination events using silent cassette *vls13* (green) as template were identified: from VR1 to VR3 (arrow, panel B) and from VR4 to VR5 (see panel A). (C) Recombination patterns obtained for variant sequences, exemplifying the following patterns: a single codon change involving any one of several possible silent cassettes (D7M5J12), a long recombination event with silent cassette 8 spanning VR2 through VR6 (D14M2B01), and 4 intermittent recombination events involving silent cassette 13 (D7M6H9).

We developed a method for visual, semi-automated analysis of the recombination events using Visual Basic macros in an Excel spreadsheet. An example is shown in Figs. 4A and 4B, in which the sequence of clone D10M8H8 (a variant isolated from a C3H/HeN mouse heart 10 days post infection) was aligned with the parental *vlsE* sequence and each of the silent cassette sequences. Each silent cassette (*vls2* through *vls16*) is represented sequentially by a different colored bar. Solid color regions represent individual codons that have undergone a sequence change and match the corresponding silent cassette in the aligned sequences. Hatched color regions are contiguous codons that match both the parental and silent cassette sequences in that part of the alignment. As shown in Figure 4B, silent cassette *vls13* (arrow) represents the most likely donor sequence due to the uninterrupted region of sequence identity spanning VR2 and VR3. Many silent cassettes match at one or more codons in this region, due to the high degree of redundancy among the *vls* at the individual codon level. However, *vls13* is the only silent cassette that provides a **contiguous** match over this entire region. It is interesting to note that *vls13* is the most likely donor sequence in two regions of the D10M8H8 sequence, separated by a short sequence that matches the parental *vlsE* sequence but not the *vls13* sequence (Figure 4A). Thus this variant may represent an example of intermittent recombination (see below).

The lengths of predicted minimum recombinations varied widely, from a single nucleotide change (*e.g.* variant D7M5J12, Figure 4C) to a region of at least 372 nucleotides (*e.g.* variant D14M2B01, Figure 4C). In some cases, especially at early time points, some variant sequences exhibited several distinct regions of gene conversion using apparently the same silent cassette source, separated by regions of unchanged parental sequence (*e.g.* variants D10M8H8 [Figure 4A] and D7M6H09, [Figure 4C]). This ‘skipping’ appears to be due to alignment of a silent cassette sequence with the *vlsE* sequence over a long distance, followed by intermittent strand invasion and replacement of the *vlsE* sequence or intermittent cleavage of a single invaded strand. These observations indicate the occurrence of so-called “intermittent recombination events” in *vlsE*. An apparent intermittent recombination event in *B. burgdorferi* had been reported previously by Knight *et al.* [34]; in this case, a sequence containing the putative Shine Delgarno sequence had been ‘skipped’ during targeted allelic exchange of the gyrase A C-terminus (*gac*) gene.

### Template-independent sequence changes

Most of the sequence changes in *vlsE* could be explained as straight-forward genetic recombinations from silent cassette sequences to *vlsE*. However, genetic changes that could not be attributed to simple gene conversion events with silent cassette sequences were found in 167 clones (Table S1 and Figure S1). These ‘template-independent’ changes encompassed a variety of genetic events ranging from single nucleotide changes, apparent illegitimate recombination events, triplet repeat expansions/contractions, or other insertions or deletions of up to 9 base pairs; they also tended to cluster in the variable regions (Figure S2). Certain codons had particularly high rates of template-independent sequence changes; for example, template-independent sequence changes of codons 73–75 were identified in 19 variant sequences isolated from 10 different animals (11% of template-independent clones). Similarly, sequence variants containing template-independent changes of codons 194–199 were isolated 23 times from 13 different animals (14% of template-independent clones). It is unclear whether these areas represent mutation ‘hotspots’ or whether mutations arising in the variable regions are more likely to be maintained due to their location. The crystal structure of VlsE [31] reveals that the variable regions (VRs) form loop structures at the membrane distal surface of VlsE while the invariant regions (IRs) form structured alpha helical bundles. Mutations arising in the IRs might destabilize the proteins. Conversely, mutations in the VRs may aid the organism in antigenic variation and be maintained preferentially.

We did recover a large number of clones showing deletions or mutations in one IR region. Codons 10–15 in IR1 contained deletions or template independent changes in 24 variants from 12 different animals (14% of template-independent clones). Sequence changes in this region included a group of 7 variants (the last clones listed in Table S1) in which related sequences differed at between 9 and 12 of 18 nt in the *vlsE1* sequence. These clones originated from skin and joint tissues of one mouse and the skin from another mouse 14 days post infection in the same experiment. These 18 nt sequences were not found elsewhere in the *B. burgdorferi* B31 genome sequence, so their source is unknown. (They are not cloning artifacts, because the PCR products were sequenced directly without cloning.) The amino acid sequence in this segment of VlsE1 is LLDKLV, whereas the corresponding variant sequences are SAVRKE, SAVQQK, SAVRQE and SADQKE. This region of IR1 is part of the α3 alpha helix in the VlsE structure [31]. Interestingly, the variant sequences preserve the alpha helical structure according to protein structure prediction programs (data not shown); thus these replacements most likely would not disrupt VlsE secondary structure.

Overall, sequences containing template-independent changes represented 15% (169 of 1,073) of *vlsE* variants, reinforcing the conclusion that they occur at a rate much higher than found in the rest of the *B. burgdorferi* genome [35]. These genetic mechanism(s) therefore may play an important role in antigenic variation of *vlsE*. Remarkably, only two of the 169 template-independent changes (a frame shift in D10M8H7 and a stop codon in SD14M4E1) represented interruptions in the open reading frame, indicating that the genetic mechanism(s) and/or selective pressure favor preservation of the full-length *vlsE* gene.

### Progressive recombination in the *vlsE* locus

**Figure 5:**
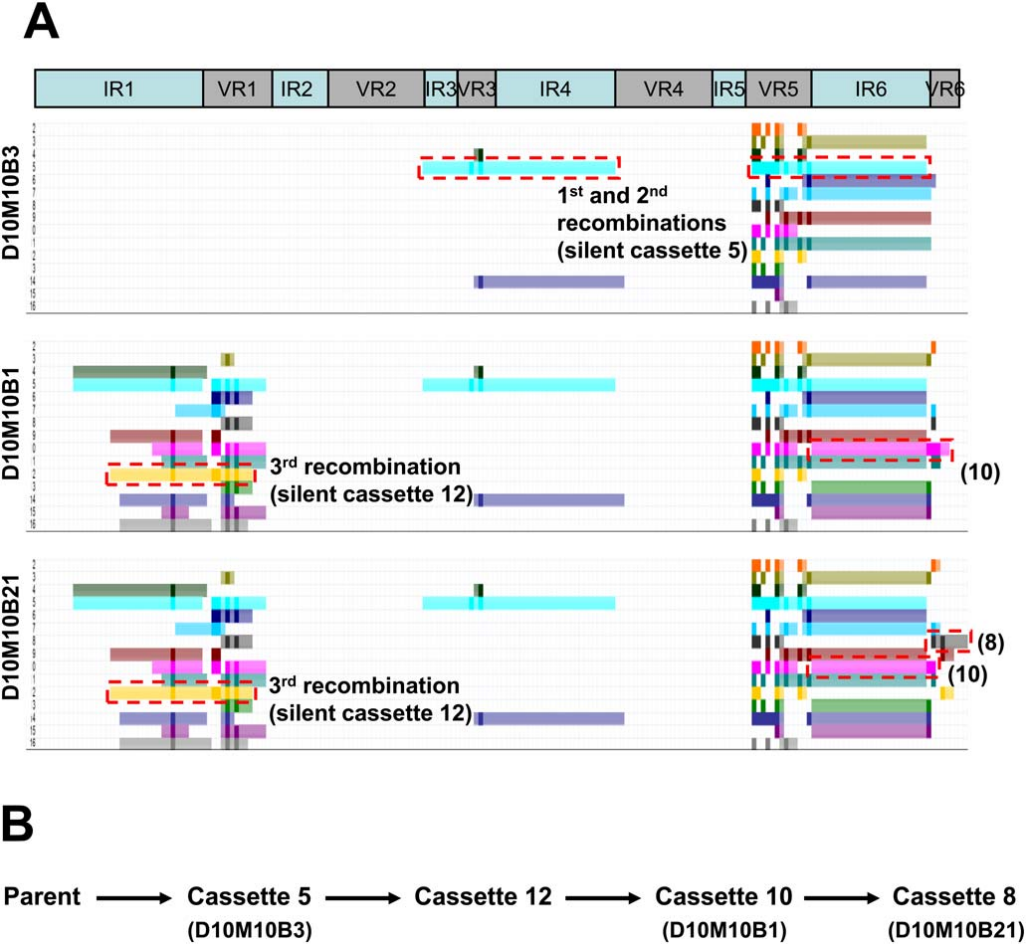
Progressive recombination in *vlsE* variant clones. (A) Schematic representation of possible recombination events for three clones derived from a single day 10 bladder culture. The shaded gray boxes indicate the variable regions (VR). Colored bars represent the maximum possible length of DNA involved in a putative recombination event for each *vls* silent cassette. Dark colored blocks within the colored bars represent observed sequence changes between *vlsE* and the variant sequence that are present in the selected silent cassette. (B) Postulated sequence of recombination events leading to the three clones.

Many *vlsE* clones appear to have undergone multiple recombination events. No direct lineage of recombinations could be identified in most cases due to the high degree of sequence variation. In rare instances, we were able to identify clones that were likely in the same ‘recombination lineage’, i.e. represented a sequential series of recombinations. Figure 5 shows an example of three clones, recovered from a single day 14 mouse bladder specimen, that have apparently undergone sequential recombination events. In panel A, the first clone, D10M10B3 was predicted to be the result of an intermittent recombination with *vls5* (the only silent cassette that contained all the sequence changes observed in both regions). The other two clones in the panel, D10M10B1 and D10M10B21, had the same sequence changes as D10M10B3 but also contain significant differences in other variable regions. Both clones contained identical sequence changes in the VR1 region that suggest a recombination event involving silent cassette 12 took place in a daughter of D10M10B3. However, D10M10B1 and D10M10B21 differ in VR6, consistent with these clones having undergone additional recombination events. Both clones contained sequence changes that are consistent with recombination with silent cassette 10 in VR6, but clone D10M10B21 exhibited additional changes in VR6 that suggest another recombination event with silent cassette 8 at some time following the recombination with *vls10*. Panel B summarizes the postulated sequence of recombination events: an initial recombination with *vls5*, followed by recombinations with *vls12* and *vls10* (in either order) and a final recombination with *vls8* in clone D10M10B21. Thus we propose that these clones represent a series of sequential recombinations.

### Increased accumulation of putative recombination events in the presence of the adaptive immune system

**Figure 6:**
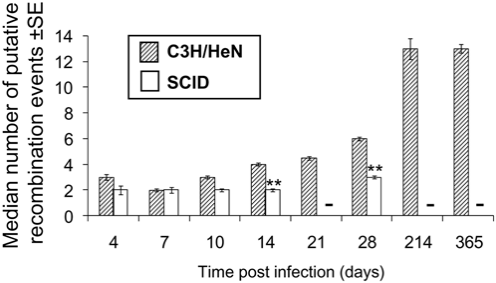
Median number of putative recombination events in variant *B. burgdorferi* clones during the time course of infection. Hatched and empty bars represent the populations of bacteria recovered from C3H/HeN or SCID mice, respectively. ** indicates a significant difference (P<0.01) between the results obtained for C3H/HeN and SCID mice for that time point, as calculated by unpaired Student's t test.

The median number of putative recombination events per clone was tabulated for each time point post infection (Figure 6). This value was found to increase significantly during the infection of immunocompetent C3H/HeN mice between day 7 and day 28 post infection (P<0.0001, Figure 6). The higher number of recombination events identified at 214 days and 365 days post infection provides further evidence that recombination continues to occur throughout the course of infection. On day 4 post infection, the number of recombination events is probably over-estimated, because several variant sequences contained intermittent recombination events (see below); by default, we considered an intermittent recombination as multiple recombination events. In contrast to the results obtained with immunocompetent mice, the number of deduced recombination events did not increase significantly between day 4 and day 28 post infection in SCID mice. These results again support a role of the adaptive immune system in the selection of clones with a higher number of putative recombination events. In a previous study, Anguita, *et al.* [36] examined a small number of *B. burgdorferi* clones and reported that the *r*ecombination at the vls locus is impaired in the absence of interferon (IFN)-γ-mediated signals. The proportion of clones that initiate *vlsE* gene conversion and the average numbers of changes per clone were lower in samples from IFN-γ receptor α-deficient mice than in wild-type mice [36]. In our study, we cannot exclude the possibility that the observed difference in the accumulation of *vlsE* variants in immunocompetent and SCID hosts is due in part to alterations in IFN-γ expression or other cytokine-mediated pathways. Other infectious agents (including *Escherichia coli*, *Mycobacterium tuberculosis* and *Trypanosoma cruzi* [37],[38],[39]) have developed diverse ways to subvert the immune system through the alteration of cytokine responses, so it is not outside the realm of possibility that *vlsE* antigenic variation is influenced by host cytokine production.

### Full-length VlsE protein expression is not required for sequence variation

The silent cassette *vls11* sequence contains a stop codon within invariable region 4 (IR4); recombination of this codon into *vlsE* would result in translational termination and a truncated polypeptide representing 63% of the full length VlsE [22],[25]. In the current study, 16 independent clones containing this stop codon in the *vlsE* sequence were isolated in the population of 1,399 clones analyzed. To determine whether a clone containing this stop codon in the *vlsE* sequence can colonize a mammalian host, two clones, 1379A and D7M5H5, were inoculated into immunocompetent C3H/HeN mice. The colonization of the mice was successful, as demonstrated by the detection of organisms in the skin at day 7 and in all tissues cultured at 28 days post infection (data not shown). We cloned sequences from the *vlsE* expression cassette to examine the ability of *B. burgdorferi* defective in full-length VlsE expression to undergo *vlsE* recombination in mice. Seven days after inoculation, bacteria recovered from back skin biopsies from 5 mice were analyzed for *vlsE* recombination. Interestingly, all 10 of the sequences analyzed still possessed the stop codon, although 50% of the clones showed changes in other parts of the *vlsE* sequence as compared to the sequence of the parental clone 1379A (data not shown) . At day 28 post-infection, *vlsE* sequences lacking the stop codon were recovered, indicating that sequences derived from the *vls11* silent cassette are capable of undergoing recombination to generate full length VlsE. Taken together, these results indicate that continuous expression of a full length VlsE protein is not required for either the successful colonization of mice or the occurrence of *vlsE* recombination. This phenomenon could be considered a form of phase variation, as occurs in the pilin expression system in *Neisseria* species [10]. These data are also consistent with several previous studies indicating that *B. burgdorferi* clones lacking either lp28-1 or the *vls* locus can disseminate and survive for short periods (<18 days) in immunocompetent mice, yet can apparently survive indefinitely in SCID mice [23],[24],[33],[40],[41].

### Predominance of short recombination events

To investigate the length of individual *vlsE* recombination events, we performed a detailed examination of clones with only one apparent event of recombination. These results comprised 126 independent clones recovered from all tested tissues from both immunocompetent and SCID mice during the time course of infection (Table 2). Variant sequences with a single event of recombination encompassed a broad range of one (*e.g.* D7M5J12) to 22 (*e.g.* D14M2B01) codon changes (Figure 4C). The recombination observed in clone D7M5J12 represented only a GGG→AAG conversion at codon 84 in the aligned sequences, and could have arisen from any of the *vls* silent cassettes containing the AAG codon at this position (*vls3*, *vls8*, *vls11*, *vls13*, *vls15 and vls16*). In this case, the minimal recombination event comprised only two nucleotides, whereas possible maximal recombination events (the range in which the variant sequence matches both the ‘recipient’ and ‘donor’ sequences on either side of the sequence change) ranged from 2 to 17 nt upstream and 21 to 37 nt downstream, depending on the silent cassette involved. Overall, the recombination event in this example involved a maximum of 25 to 48 nt of DNA, indicating that *vlsE* recombination can take place in a very small region. At the other end of the spectrum, the putative recombination event with *vls4* in clone D14M2B01 (Figure 4C) encompassed a minimum of 349 nt and a maximum of 423 nt of donor sequence, with 64 nt and 10 nt of sequence identity flanking the region of sequence change on the upstream and downstream ends, respectively. Thus the *vlsE* recombination system appears to promote both minuscule and long recombination events within the cassette region.

**Table 2:** Number of *vlsE* sequences analyzed exhibiting a putative single recombination event.

Days post infection	No. of sequences									
	C3H/HeN					SCID				
	Bladder	Heart	Joint	Ear	Skin	Bladder	Heart	Joint	Ear	Skin
4 days					1*					
7 days	11	6	18				1	4		
10 days	11	7	13		9	3	5	1	3	
14 days			2		3	5	7	5	1	1^§^*
28 days	1*					3^§^*	5^§^*	2^§^*		3^§^*

The description and key are the same as in Table 1.

**Figure 7:**
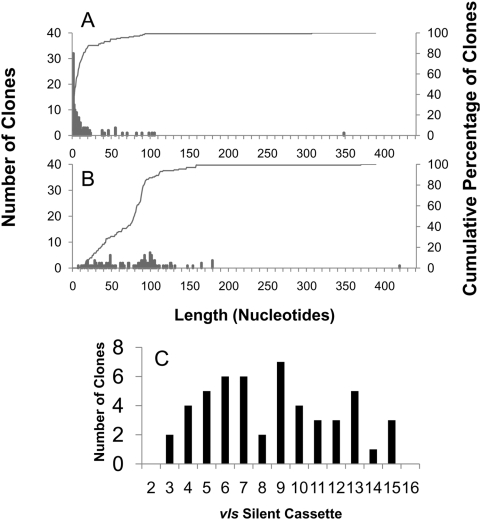
Lengths of minimum and maximum predicted recombination events in 126 clones identified as having a single, well-defined recombination event. Histograms of the deduced (A) minimum and (B) maximum lengths of recombination are depicted as bars; the cumulative percentage of clones having predicted minimal or maximal recombination lengths≤the length shown are represented as lines. Panel C represents the putative silent cassette usage in clones with a single recombination event in which the silent cassette source could be determined unambiguously.

A subset of 126 *vlsE* variants was identified that appeared to represent ‘templated’ single recombination events (Table S2). All of the sequence changes in this group had corresponding template sequences in one or more silent cassettes, and most had regions of homology with both the ‘donor’ and ‘recipient’ sequences both upstream and downstream from the sequence change (*e.g.* D7M5J12, Figure 4C). The majority of these clones (70 of 126, or 55%) exhibited a minimum region of recombination of 1 to 5 nucleotides (Figure 7A) Amazingly, 33 of 126 (26%) had only a single nucleotide change (Figure 7A). These most likely represent templated gene conversion events, because they occur at a much higher frequency than that of template-independent single nucleotide sequence changes (76 of 1,073 sequences examined, or 7%) (Table S1).

An additional 28 clones (22%) exhibited minimal recombination events of 6 to 14 nt, whereas only 28 clones (21%) had minimal recombinations ≥15 nt. In contrast, the lengths of the predicted maximum recombination events were more widely distributed between 7 to 419 nucleotides (Figure 7B). (In this analysis, the maximal recombination event was based on the longest region of sequence identity if more than one silent cassette could have served as the ‘donor’ for the recombination event.) These results indicate that the length of DNA that is utilized during the recombination process is highly variable, but tends to include a short region of nonhomologous DNA. The observed median minimal recombination (±S.E.) was 5±0.27 nt, whereas the median maximal recombination (±S.E.) was 89±0.37 nt. The difference between minimal and maximal recombinations reflects the high homology between the silent cassette sequences and the *vlsE* cassette region. Thus, each round of *vlsE* gene conversion (*i.e.* each *vlsE* recombination event) often introduces only one or two amino acid changes in each variant protein sequence, although a much larger region may be involved in each recombination event. To examine this mechanism further, the base changes occurring in 33 well-defined ‘templated’ single nucleotide changes (proposed gene conversion events) were compared with 76 ‘template-independent’ changes (Table S3). While the proportion of nucleotide conversions were similar overall, C→A transversions were favored in the templated group, and C→G transversions were predominant in the template-independent group; this result implies that different mechanisms are operative in the two groups. While we cannot determine conclusively whether single nucleotide changes were introduced by genetic recombination or hypermutation as previously proposed by Sung *et al.* [35], our study indicates that most sequence changes in *vlsE* result from gene conversion events between the *vls* silent cassettes and the *vlse1* expression cassette.

### Silent cassette usage as recombination template

The putative silent cassette usage was determined for the sequence of clones showing a single, non-ambiguous recombination event (Figure 7C). We observed that some silent cassettes, including *vls6*, *vls7*, *vls9*, appeared to be used more often than the others. Although it had been proposed previously that the 17 bp direct repeat sequences present at the 5′ and 3′ ends of each silent cassette are involved in *vlsE* recombination [22], those silent cassettes with poorly conserved direct repeats (e.g. cassettes 10 and 11) are used during *vlsE* variation (Figure 7C). In the population of clones with a single recombination event, no clones were identified in which silent cassettes *vls2*, *vls14* and *vls16* were used as template for recombination. To extend this analysis, we also identified well-separated, unambiguous recombination events in all 1,073 clones with *vlsE* variations, including those with multiple recombination events. In this extended group, examples where *vls2*, *vls14* or *vls16* were unambiguously used as template were observed (data not shown). These results indicates that any region of any silent cassette can be use as template, although the silent cassettes present in the central part of the silent cassette locus tend to be used more frequently.

### Distribution of sequence variations within the *vlsE* cassette region

**Figure 8:**
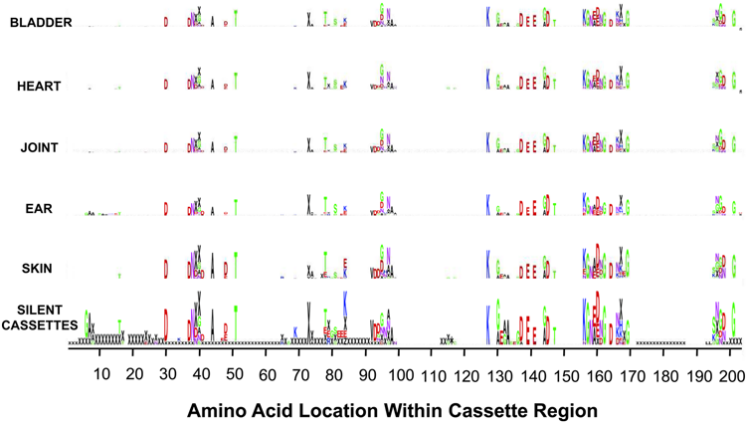
The locations and amino acid utilization of deduced VlsE amino acid changes paralleled the changes predicted by the sequence differences between *vlsE1* and the silent cassettes. The distribution of amino acid changes found in the variant sequences from different infected tissues were depicted as sequence logo patterns using the program WebLogo [54]. The height of the letter is proportional to the frequency of changes. The letter “x” indicates a 3-nt indel, and the asterisk a stop codon. The panels for bladder, heart, joint, ear, and skin represent the observed changes in the variant sequences recovered from the respective tissue at all time points during *B. burgdorferi* infection of C3H/HeN or SCID mice. The silent cassette panel represents the relative probability of a given amino acid change at each position of *vlsE1*, based on the amino acids encoded by the silent cassette sequences at each position in the *vlsE1*/silent cassette alignment.

In our study, we analyzed the location of sequence changes within the *vlsE* cassette region during the time course of infection. No evidence of a recombination ‘program’ in which recombinations involved certain variable regions earlier than others was observed (data not shown). We also checked each individual variant amino acid sequence to determine if a specific VlsE protein sequence can be linked with a specific tissue tropism. In the relapsing fever organism *Borrelia turicatae*, the variable large protein/variable small protein (Vlp/Vsp) antigenic variation system influences tissue tropism as well as immune evasion [42],[43]. For example, *B. turicatae* expressing VspA are neurotropic while those expressing VspB achieve higher concentrations in the blood in a mouse model [42],[43]. In our study, there were no obvious differences in the amino acid sequence changes observed in different tissues (Figure 8). These results suggest that the *vlsE* gene conversion system is primarily involved in immune evasion rather than tissue tropism. Interestingly, we were also able to identify 5 pairs of clones presenting the exact same variant sequence in different tissues of the same mouse (e.g. D28M2BX1 from bladder and D28M2HX6 from heart); an additional 33 pairs of identical variants in different tissues were identified in other mice. These findings verify the occurrence of dissemination of variant clones in individual mice.

### Conclusion

The *vls* antigenic variation system is an example of segmental gene conversion, which is also found in the *N. gonorrhoeae pilE* system [44], the *A. marginale msp2* system [13], and the *Babesia bovis ves1α* system [45]. In each of these systems, a set of silent gene segments serves as the source of the ‘donor’ sequence, but the donor site remains unaltered in progeny following the recombination. Also, the recombination events occur at a high rate within the target gene, indicating that special mechanisms facilitate unidirectional genetic change in the target site (but not the donor sites). Another unusual property shared by segmental gene conversion mechanisms is that the recombination is ‘unanchored’ within the target gene, i.e. it does not start or stop at a certain sequence. The relapsing fever antigenic variation system is different in this aspect, in that most gene conversion recombination events occur at specific upstream and downstream homology sequences [16]. Our data also indicated that very short recombinations occur in the *vls* system, and that long flanking regions of sequence identity between the donor and recipient sequences are not required. Indeed, in our analysis of probable single recombination events, there were examples where there was no sequence identity on one end or the other of the recombination (e.g. clones D7M3B12, D10M9H6, and D10M7J4). The day 7 joint isolate D7M2J05 (data not shown) exemplified clones with short segments of sequence identity at both ends of the recombination, with regions of identity of only 2 nt and 6 nt upstream and downstream of an 11 nt region of sequence replacement (from cassette 10). These results indicate that the *vls* recombination system requires very little sequence identity to initiate the recombination event. In this regard, the *vls* system appears to be similar to the *N. gonorrhoeae pilE* system, in which sequence changes ranging in size from as short as 1 nt to as long as 200 nt have been observed; in addition, over 50% of the recombinations are 15 nt or less [44],[46],[47]. In no case, however, has illegitimate recombination into nonhomologous sites been observed in *vlsE* (or *pilE*), demonstrating that some extent of sequence complementarity and alignment is needed to ‘nucleate’ the recombination event.

The mechanisms involved in segmental gene conversion during antigenic variation are not well understood. *pilE* sequence variation is RecA-dependent [48], and appears to involve circular intermediates that are derived from *pilS* silent cassette sequences [49]. We propose that a *vls* silent cassette sequence (in the form of lp28-1, a separate episomal DNA copy, or possibly even an RNA copy) undergoes strand invasion, displacing the parental strain. This process requires very short regions of sequence identity and could be facilitated by a DNA-binding protein or endonuclease to nick the recipient and/or donor DNA, although the lack of specificity in terms of the site of sequence change suggests that these activities would not be very site-specific. The strand invasion also appears to be terminated in a non-specific manner, in that the lengths of recombination were variable (although predominantly short). Termination may not require a region of sequence identity, in that there were examples where no region of sequence identity was found at one end of the *vlsE* sequence change. We believe that as yet unidentified mediators of *vlsE* recombination are induced or activated during mammalian infection, as evidenced by rapid occurrence of *vlsE* sequence changes during mammalian infection and the lack of detectable sequence variation during in vitro culture or tick infection. (Alternatively, an inhibitor of *vlsE* recombination could be repressed or inactivated during mouse infection; however, this scenario seems unlikely in that *vlsE* recombination has not been observed in *E. coli* transformed with constructs containing *vlsE* and an adjacent region of the silent cassettes [S. J. N. and J. K. Howell, unpublished data].) Study of this phenomenon and its *cis-* and *trans*-acting mediators would be aided greatly by the identification of conditions that activate *vlsE* recombination in vitro, or *vls* shuttle constructs that undergo recombination in *B. burgdorferi* and can be genetically manipulated to permit the identification of *cis-*acting elements.

Antigenic variation and phase variation in bacterial surface proteins are common and have been shown to contribute to avoidance of adaptive immune responses, to tissue tropism, or to the pathogenesis process (e.g. altered adherence properties). Our studies provide direct *in vivo* evidence of the function of gene conversion of the Borrelial VlsE lipoprotein. In wild-type mice (in comparison with SCID mice), clones having the parental *vlsE* sequence persist for a shorter period; in addition, *vlsE* codon changes and recombination events accumulate more rapidly. These data indicate that variants are selected in immunocompetent mice, most likely due to antibodies specific for the variable regions of VlsE [32]. Similar results have been observed in the Vlp/Vsp antigenic variation system of relapsing fever Borrelia [50] and the Vsa phase variation system in *Mycoplasma pulmonis* [12]. The adaptive immune system thus acts as a selective force, killing clones with less variation but not eliminating clones with more extensive variation (and hence antigenic differences). In this study and as previously observed by Zhang and Norris [25], the presence or absence of the adaptive immune system is not required to induce the *vlsE* gene conversion mechanism. However, we cannot rule out the possibility that the adaptive immune system can directly affect the kinetics of the ongoing process of *vlsE* recombination, *i.e.* that the bacteria exhibit increased recombination under the influence of immune pressure (*e.g.* production of specific antibody or cytokines) [36]. Indeed, *vlsE* expression is increased under the influence of the immune pressure, specifically when functional B cells are present [51]. An interesting experiment would be to follow the rate of *vlsE* variant accumulation during the time course of infection of immunocompetent or SCID mice previously immunized with recombinant VlsE protein. An additional finding was that the production of a stable VlsE protein is not required for the *vlsE* gene conversion process to occur. Any silent cassette (and any region thereof) can be involved in a recombination event, and a variety of apparent template-independent genetic changes contributed to sequence variation. The recombination events are evenly distributed throughout the *vlsE* cassette region and exhibit no apparent bias for particular regions. Furthermore, no VlsE motif was associated with infection of a specific tissue site. The degree of variation observed indicates that the *vlsE* recombination system is one of the most robust antigenic variation systems found in pathogens.

## Materials and Methods

### Bacterial strains and cultures

The high-infectivity *B. burgdorferi* B31 clones 5A3 (B31-5A3, lacking plasmids lp28-2 and lp56) and 5A4 (B31-5A4, containing all plasmids) were isolated previously from low-passage strain B31 [27]. Small quantities were removed from the surface of frozen stocks by scraping with sterile 1 ml pipet tips and were inoculated into 6 ml of BSKII medium [52]. Cultures used in this study had undergone no more than two passages since clone isolation, thus minimizing the likelihood of plasmid loss.

### Animal studies

All research involving animals was approved by the Animal Welfare Committee of the University of Texas Health Science Center at Houston. Eight-week-old, female C3H/HeN mice (Harlan, Indianapolis, IN), C3H/HeN severe combined immunodeficiency (SCID) mice (Harlan) and CB-17 SCID mice (Charles River Laboratories, Wilmington, MA) were housed in microisolator cages and provided with antibiotic-free food and water. For mouse inoculation, frozen stocks of low passage *B. burgdorferi* strains were cultured in BSK II medium [52] at 37°C in 3% CO_2_ until the mid-log phase of growth. The cultures were diluted in BSK II medium to a concentration of 10^6^ bacteria/ml as determined by dark-field microscopy, and 0.1 ml (10^5^ organisms) was injected subcutaneously at the base of the tail. Groups of 4 to 6 mice were sacrificed on 4, 7, 10, 14 and 28 days post infection, and samples from tissue sites (bladder, heart, joint, ear and skin) were acquired under aseptic conditions and cultured in 6 ml of BSK II broth with an antibiotic mixture to reduce the occurrence of microbial contamination (Sigma Aldrich; 50 µg/ml rifampin, 20 µg/ml phosphomicin and 2.5 µg/ml amphotericin B). After 7 days, the cultures were checked for growth, diluted, and subsurface plated in BSKII-agarose medium to obtain individual clones as described previously [53].

### Amplification and sequencing of *vlsE* cassette region

Well-isolated colonies from BSKII-agarose plates were inoculated in BSK II medium with antibiotics and cultured for 4 days prior to use as PCR templates (≈10^4^ cells per reaction). Alternatively, agarose plugs containing individual colonies were added directly to the PCR reaction. The *vlsE* cassette region of each clone was amplified by PCR using the Phusion High-Fidelity DNA Polymerase (New England BioLabs, Ipswich, MA) and *vlsE* primers 4066 and 4120 as described previously [25]. The PCR products were purified and sequenced on both strands at the High-Throughput Genomics Unit (Department of Genome Sciences, University of Washington , Seattle), utilizing the same primers used for the amplification. The PCR products were sequenced directly (without cloning the products) to minimize the effects of sequence errors due to PCR infidelity. The chromatographs corresponding to each DNA sequence were examined individually to verify the quality of the sequence data, and each sequence difference (in comparison to the parental *vlsE1* sequence) was checked for sequence accuracy. Variant clones that originated from the same tissue specimen and had identical sequences were considered siblings but were treated separately in this analysis.

### *vlsE* variant sequence analysis

The B31 parental *vlsE* (allele *vlsE1*), *vls* silent cassettes, and all of the *vlsE* variants sequences presented in this study are contained in GenBank entries U76406, U76405, EU484573–EU485396, EU485400–EU485403, EU485405–EU485714, EU485716–EU485724, EU485726–EU48748, and EU485750–EU485984; a list of the clone numbers and the corresponding GenBank accession numbers is at http://www.uth.tmc.edu/pathology/borrelia/. Most clone numbers are in the following format: S = SCID mouse infection; D4 = 4 days post infection; M3 = mouse 3; B, E, H, J, S = bladder, ear, heart, tibiotarsal joint, and skin, respectively; number and/or letter designations = individual clone from that animal and tissue. An ‘X’ indicates that a colony PCR product was sequenced, and no *B. burgdorferi* culture was retained. Infecting clone refers to either B31 5A3 (lacking plasmids lp28-2 and lp56) or B31 5A4 (containing all plasmids).

The DNA sequences of the parental *vlsE* cassette region (*vls1*) and the silent cassettes (*vls2* to *vls16*) were aligned manually to match the arrangement in Figure 3 of Zhang *et al.* [22], and their sequences were formatted into codons (corresponding to *vlsE* codons). Indels were recorded using the letters “OOO” as a place marker. The aligned sequences were inserted into a Microsoft® Excel spreadsheet (one codon per cell), creating the template used to analyze *vlsE* variant sequences. Each *vlsE* variant sequence was codon-formatted, trimmed, and optimally aligned with *vls1* (using the ClustalW multiple alignment algorithm embedded in the Bioedit software [http://www.mbio.ncsu.edu/BioEdit/BioEdit.html] (followed by manual adjustments) prior to analysis. The sequences were then compared to the parental *vls1* sequence and the silent cassette sequences using a set of macros written using Microsoft® Visual Basic for Applications. The Excel template and macros may be obtained by contacting the authors.

The nucleotide and deduced amino acid sequences of each variant were compared computationally to both *vls1* and the silent cassette nucleotide and predicted amino acid sequences. We first analyzed the overall number of codon differences between *vls1* and the variant sequence. The codon sequence for each individual observed difference was compared to the sequences present among the silent cassettes at the same position, thus determining the putative silent cassette source(s) of any non-parental codon found in a given variant sequence. By combining the location and the possible silent cassette sources for each change in a variant sequence, we were able to identify regions of sequence variation and to propose putative events of recombination as well as the silent cassettes potentially used as templates. For each region of sequence variation, the minimal deduced regions of recombination were defined as groups of contiguous codons differing from the parental sequence and matching a silent cassette sequence, whereas the maximal deduced regions of recombination included all contiguous codons in either direction in which the variant sequence matched both the parental and silent cassette sequences. The possible recombination events were thus determined computationally and portrayed graphically by the Excel™ spreadsheet, as exemplified in Figure 4. In cases where more than one silent cassette could serve as the template for a recombination event, the silent cassette showing the longest maximal recombination pattern was selected as the possible template.

### Positional changes in *vlsE* variants with time

By comparing the silent cassette sequences and the *vls1* sequence, we determined the probability of change at each codon in *vls1*. Each *vlsE* variant sequence was then compared to *vls1* to determine the number and position of codon and amino acid differences in that variant. The results obtained for variant sequences at a given time post infection were analyzed together, and the total number of differences at each position was calculated, normalized for the number of variants, and compared with the probability of change at each position in *vls1*. The deduced amino acid changes occurring at each position were also compared to probability data obtained from *vlsE1*/silent cassette comparisons and displayed using the program WebLogo [54].

### Statistical analyses

Statistics were performed in Microsoft® Excel using the unpaired Student's t test.

## Supporting Information

Table S1 *B. burgdorferi vlsE* variant clones with template-independent sequence changes.
(0.12 MB PDF)

Table S2 *B. burgdorferi vlsE* variant clones with sequences consistent with a single recombination event.
(0.11 MB PDF)

Table S3 Comparison of the base changes occurring in ‘templated’ vs. ‘template-independent’ single nucleotide changes in *vlsE* variants.
(0.08 MB PDF)

Figure S1 D28M1HX5 as an example of a *vlsE* variant with templated and template-independent sequence modifications. Possible involvement of silent cassette sequences *vls2–vls16* in *vlsE* gene conversion events are shown by the colored bars, as described in the legend for Fig. 4. Variant D28M1HX5 contains an template-independent variations at codons 147 and 165. This mismatch to any *vls* silent cassette is indicated by a 0 in the *vlsE* match row as well as by the lack of color fill in the graphic column of those codons. Variant D28M1HX5 has apparently undergone multiple templated recombination events, with the most likely ‘donor’ sequences being *vls6* (minimal recombination event = codons 37–64), either *vls3* or *vls4* (98–127), *vls7* or *vls10* (141–144), *vls8* (156–162), and *vls3* (169–201).
(0.09 MB XLS)

Figure S2 Locations of template-independent sequence variation. The X axis numbers represent the codon number of each amino acid in *vlsE* as presented in Figure 3 of [22]. The dark blue bars represent the number of variants recovered at each codon. The black line represents a 3 point moving average. In the diagram below the graph, the light areas indicate the locations of the invariable regions of *vlsE* while the dark areas indicate the positions of the variable regions of *vlsE*.
(0.07 MB PDF)
