# Supplementary material for: Detailed Analysis of Sequence Changes Occurring during vlsE Antigenic Variation in the Mouse Model of Borrelia burgdorferi Infection
Source: PLoS Pathog. 2009 Feb 13;5(2):e1000293. doi: 10.1371/journal.ppat.1000293 (PMC2632889; doi:10.1371/journal.ppat.1000293)
Supplement: Table S1 — B. burgdorferi vlsE variant clones with template-independent sequence changes. (0.12 MB PDF) [file ppat.1000293.s001.pdf]

Table S1. *B. burgdorferi vlsE* variant clones with template-independent sequence changes[a]

Clone Name	No. codons affected	Nt differences	Codon(s)[b]	Sequence change(s)[c]	NCBI Accession Number
SD28M1HX4	3	-9	163-165	GCGGAGTTT → OOOOOOOOO; 9 bp deletion	EU485914
D10M8B2	3	-9	42-44	GCAAAGGTT → OOOOOOOOO; 9 bp deletion	EU484884
D7M5H03	1	-6	159-160	GGGGGT → OOOOOO; 6 bp deletion, same as D7M5H04	EU484717
D7M5H04	1	-6	159-160	GGGGGT → OOOOOO; 6 bp deletion, same as D7M5H03	EU484718
D7M5H08	1	-6	159-160	GGGGGT → OOOOOO; 6 bp deletion, same as D7M5H13, D7M5H09	EU484722
D7M5H09	2	-6	159-160	GGGGGT → OOOOOO; 6 bp deletion, same as D7M5H08, D7M5H13	EU484723
D7M5H13	2	-6	159-160	GGGGGT → OOOOOO; 6 bp deletion, same as D7M5H08, D7M5H09	EU484752
D7M5H05	2	-6	163-164	GCGGAG → OOOOOO; 6 bp deletion	EU484719
D10M9B4	2	-6	41-42	GCTGCA → OOOOOO; 6 bp deletion	EU484899
SD28M3BX1B	1	-3	75	GCT → OOO; 3 bp deletion within GCTGCTGCT tract	EU485873
D10M8H7	1	-1	100	1 bp deletion; frameshift	EU484948
D28M2HX31	1	1	8	AGC → CGC	EU485432
D10M7J12	1	1	9	GAG → GGG	EU484996
D14M1H10	1	1	10	TTG → TCG	EU485130
D14M4S1	1	1	10	TTG → TTT	EU485274
D10M11H10	1	1	13	AAG → AAC	EU484914
D10M8B7	1	1	13	AAG → AAC	EU484889
D28M3B03	1	1	13	AAG → AAC	EU485356
D10M10B4	1	1	15	GTA → GAA	EU484850
D10M11H7	1	1	15	GTA →GAA	EU484922
D10M11H9	1	1	41	GAT → GAG	EU484924
D10M7J2	1	1	73	GCT → GTT	EU484997
D10M8H5	1	1	73	GCT → GTT	EU484946
D28M2HX6B	1	1	73	GCT → GTT	EU485437
D7M2H15	1	1	73	GCT → GTT	EU484741
D14M5B9	1	1	73	GCT → GTT	EU485102
D7M3H18	1	1	73	GCT → GTT	EU484745
D14M3J11	1	1	74	GTT → GGT	EU485215
D14M3J12	1	1	74	GTT → GCT	EU485216
D7M2H14	1	1	74	GTT → GGT	EU484740
D10M8B8	1	1	75	GCT → GTT	EU484890
D14M3S1	1	1	75	GCT → GTT	EU485263
D14M3S11	1	1	75	GCT → GTT	EU485265
SD10M2JX3	1	1	75	GCT → GTT	EU485665
SD10M3HX1	1	1	75	GCT → GTT	EU485628
D10M11B2	1	1	80	GAG → GGG	EU484856
D10M8S3	1	1	80	GAG →GGG	EU485064
D10M9H12	1	1	80	GAG → GGG, same as D10M9H2	EU484952
D10M9H2	1	1	80	GAG → GGG, same as D10M9H12	EU484953
D10M9H8	1	1	80	GAG → GGG	EU484960
D14M6S9	1	1	84	GGG → AGG	EU485254

Clone Name	No. codons affected	Nt differences	Codon(s)[b]	Sequence change(s)[c]	NCBI Accession Number
1501C bladder	1	1	97	CAT → GAT	EU485526
SD28M2HX3	1	1	97	CAT → GAT	EU485922
D14M6S10	1	1	98	GCT → GGT	EU485249
D7M2B03	1	1	99	AAT → CAT	EU484626
D7M5J10	1	1	99	AAT → CAT	EU484796
D7M6H05	1	1	99	AAT → CAT	EU484728
D7M6H07	1	1	99	AAT → CAT	EU484730
D28M1HX7	1	1	123	AGT → AGC	EU485402
SD28M2JX11	1	1	127	ACG → ATG	EU485948
D10M11J12	1	1	132	GGT → GAT	EU484974
D10M11J5	1	1	132	GGT → GAT	EU484978
D10M7S3	1	1	132	GGT → GAT	EU485054
SD28M1HX6	1	1	137	GAG → GAC	EU485916
D7M3B07	1	1	144	GAG → GCG	EU484640
D28M2HX32	1	1	149	CCG → CCC	EU485433
SD28M1HX12	1	1	157	AAA → GAA	EU485907
D10M10B12B	1	1	159	GGG → GAT	EU484844
D7M2B09	1	1	159	GGG → GGT	EU484632
D14M1H11	1	1	166	GGT → AGT	EU485131
M4E4A	1	1	166	GGT → AGT	EU485483
SD14M4E1	1	1	166	AAG → TAG; stop codon	EU485820
SD28M1HX18	1	1	167	CAG → CTG	EU485910
D28M2HX30	1	1	169	GAG → AAG	EU485431
SD10M3JX4B	1	1	170	ATG →AAG	EU485671
D14M1H9	1	1	175	CAG → CAT	EU485135
D10M10J9	1	1	185	ATG → AGG	EU484970
1251C skin	1	1	194	AAG → AGG	EU485521
D14M5H3	1	1	194	AAG → AAT	EU485144
D10M11H2	1	1	195	AAG → GAG	EU484917
D10M11H5	1	1	195	AAG → GAG	EU484920
D14M5H2	1	1	195	GAT → GGT	EU485143
D14M6J8	1	1	195	AAG → AGG	EU485203
1302A skin	1	1	196	GAG → GAT	EU485292
D10M11H11	1	1	196	GAT → GAG	EU484915
D14M2J9	1	1	196	GAT → GAG	EU485181
M8E4A	1	1	196	GAT → GAG	EU485485
D28M2JX17	1	1	199	GAG → GGG, same as D28M2JX7	EU485472
D28M2JX6	1	1	199	GAG → GGG	EU485477
D28M2JX7	1	1	199	GAG → GGG, same as D28M2JX7	EU485478
D14M1H12	1	1	201	GAG → AAG	EU485163
D10M10J8	1	1	202	AAG →GAG	EU484969
D14M1S2	1	1	202	AAG → GAG	EU485255
D28M1J10	1	1	202	AAG → AAC	EU485449
SD28M1H01	1	1	202	AAG → ACG	EU485902
D14M2B8	1	1	203	GAT → GCT	EU485117
D28M1J06	1	1	202	AAG → AAA	EU485447
D10M12B1	1	2	13	AAG → CAC	EU484863
SD14M2H6	1	2	40	GAT →ACT	EU485739
D14M5H4	1	2	79	GGG → AAG	EU485145
1255C skin	2	2	96, 156	GCT → GGT; GAT → GAG	EU485523
1302B skin	1	2	96	GCT → CAT	EU485293
D28M2BX4	1	2	166	GGT → GAG	EU485383
SD14M2E1	1	2	167	CAG → GAT	EU485838

Clone Name	No. codons affected	Nt differences	Codon(s)[b]	Sequence change(s)[c]	NCBI Accession Number
D10M11B6	1	2	196	GAT → GTA	EU484860
M2B4A	1	2	196	GAT→AAG	EU485361
D14M2H2	2	2	13, 15	AAGCTGGTA → AACCTGGAA	EU485738
SD28M1HX19	2	2	141, 169	AAG → CAG; GAG → AAG	EU485911
D14M1B12	2	2	14-15	CTGGTA → CAGGAA	EU485083
SD14M1E2	2	2	15, 132	GTA →GAA, GGT → GAT	EU485827
1375B SKIN	3	2	160, 165	GGT → AGT, TTT → TTG	EU485289
D28M1HX1	2	2	199, 202	GAG → GAT, AAG → AAA	EU485409
D14M5H11	2	2	200, 203	AAA → GAA; GCT → GAT	EU485141
D14M5S9	2	2	201, 203	GAG → AAG; GCT → GAT	EU485247
D14M4H3	2	2	3, 10	GCT → CCT; TTC → TTT	EU485743
D14M2H9	2	2	8, 13	AGC → AGT; AAG → AGC	EU485760
1500B bladder	2	2	96-97	GCTCAT → GGTGAT	EU485525
1502D skin	2	3	73, 167	GCT→TTT; CAG → GAT	EU485528
D21M2S05	3	3	199-201	GAGAAAGAG → GATAAGAAG	EU485323
1417A HEART	1	3	41	GCT → OOO	EU485388
1417C HEART	1	3	41	GCT → OOO	EU485390
SD10M3HX4B	1	3	97	apparent duplication of codon 96 (GCT)	EU485638
D28M2HX24	1	3	98	OOO → GTC	EU485427
SD14M4EX3	1	3	98	OOO → CAT	EU485844
1498A bladder	1	3	99	OOO → CAT	EU485524
SD28M2HX1	1	3	99	OOO → CAT	EU485919
1388B skin	1	3	132	OOO → GAT	EU485299
D21M2S06A	1	3	132	OOO → GAT	EU485333
D28M1B02	1	3	132	OOO → GAT	EU485348
1394D skin	1	3	161	OOO → GCT	EU485502
1414B SKIN	1	3	161	OOO → GGG	EU485969
D28M1S01	1	3	161	OOO → GCT	EU485978
D28M1S10	1	3	161	OOO → GGT	EU485512
D28M2BX1	1	3	161	OOO → GGT	EU485866
D28M2BX18	1	3	161	OOO → GGT	EU485380
D28M2HX6	1	3	161	OOO → GGG	EU485436
SD28M3S K	1	3	161	OOO → GGG	EU485981
D28M1B10B	1	3	162	OOO → GGC	EU485367
D28M1H12	1	3	162	OOO → GCT	EU485395
D14M3B11	1	3	195	OOO → GAT, 3 bp insertion	EU485119
D28M1J01	1	3	197	OOO → GAG	EU485445
D10M11B5	3	3	11, 15, 51	TTG → TCG; GTA → GAA; AAG → AGG	EU484859
D28M1HX5	3	3	138,147, 165	CAG → GAG; AAA → GAA; TTT → TTG	EU485401
SD28M1HX17	2	3	140, 169	GGA → GAA; GAG → GAA	EU485909
D28M1H11	2	3	144, 196	GAG → GCG; GAT → AGT	EU485394
D28M2BX10	2	3	144, 199	GAG → GCG; GAG → TAA	EU485375
D10M10B12	2	3	159, 190	GGG → GAT; AAG → GAG	EU484843
D21M2S06	3	3	26, 136, 196	TCA → TCG; GCT → GAT; GAT → GAG	EU485324
1379C skin	1	3	40, 42, 44	GAT → GGT; GCA → GCG; GTT → GCT	EU485849
1502F skin	2	3	73, 167	GCT → GTT; CAG → GAT	EU485530
1502G skin	2	3	73, 167	GCT → GTT; CAG → GAT	EU485531
D14M1S7	3	3	78, 97, 203	ACA → ACT; CAT → GAT; GCT → GAT	EU485257
SD14M2E5	2	4	157, 167	AAA → GGA; CAG → TTG	EU485818
SD14M1HX7	4	4	120, 124, 126-127	CAG → AAG; GCG →GTG; GTT →GCG; ACG →GCG	EU485757
SD14M1E2	2	4	15, 132	GTA →GAA; OOO → GAT	EU485827
SD14M2JX3	2	4	15, 99	GTA → GAA; OOO → CAT	EU485792

Clone Name	No. codons affected	Nt differences	Codon(s)[b]	Sequence change(s)[c]	NCBI Accession Number
D10M8S1	2	4	165, 167	TTT → AAT; CAG → GCG	EU485061
D14M1B8	2	4	166, 199	GGT → OOO; GAG → GAT	EU485087
SD10M4BX6	2	4	195, 199	OOO → AAT; GAG → GAT	EU485626
D14M5S2	2	4	75, 78	GCT → GTT; AAA → GCT	EU485241
D7M6H10	3	5	132, 158-159	OOO → GAT; GATGGG → GAGGGT	EU484732
SD10M2JX4	3	5	15, 159-160	GTA → GAA; GGGGGT → GACGAC	EU485666
SD14M3B2	2	5	157, 166	AAA → TCT; GGT → TGA	
D28M2B04	2	5	158, 161	GAT → AAA; OOO → GGT	EU485371
D14M5S3	4	5	199, 201-203	GAG → AAA; GAGAAGGCT → AAGAATGAT	EU485242
1394C skin	2	5	77-78	GCTAAA → GAAOOO	EU485501
D28M2HX19	4	6	108, 138, 144, 162	GCG → GCT; CAG → CAT; GAG → GCG; OOO → AAT	EU485422
D14M1B2	3	6	13-15	AAGCTGGTA → GCGAAAGAA	EU485084
D28M1E9	2	6	162, 197	OOO → GCT; OOO → AAG	EU485491
D14M2S10	3	7	96, 98, 99	GCT → GGT; OOOOOO → AATGCCT	EU485259
SD10M3HX3	5	8	14, 26-29	GTA → GAA; TCAAGTGGTACT → ATTAAGGGAGCT	EU485637
D14M1J5	6	12	10 to 15	TTGTTGGATAAGCTGGTA → TCTGCGGTTCGCAAAGAA	EU485773
D14M1J6	6	12	10 to 15	TTGTTGGATAAGCTGGTA → TCTGCGGTTCGCAAAGAA	EU485774
D14M1S1	6	9	10 to 15	TTGTTGGATAAGCTGGTA → TCTGCGGTTCAGCAAAAA	EU485227
D14M1S4	6	11	10 to 15	TTGTTGGATAAGCTGGTA → TCTGCGGTTCGCCAAGAA	EU485228
D14M1S6	6	12	10 to 15	TTGTTGGATAAGCTGGTA → TCTGCGGTTCGCAAAGAA	EU485229
D14M1S8	5	9	10-11, 13-15	TTGTTGGATAAGCTGGTA → TCTGCGGATCAGAAAGAA	EU485230
D14M2H8	6	10	10 to 15	TTGTTGGATAAGCTGGTA → TCTGCGGTTCGCAAGGAA	EU485741

[a] An example of the analysis of a *B.burgdorferi vlsE* variant with a template-independent sequence modification is presented in Fig. S1.

[b] Codon numbering corresponds to the location in the aligned cassette sequences as first described in Fig. 3 of [22] and also depicted in Fig. S1 of this article.

[c] The sequences of the parental clone and of the recovered variant with template-independent change are separated by an arrow. In clones in which there are more than one template-independent change, they are provided in the same order as shown in the Codon(s) column. Relative deletions/insertions are indicated as "O".
