# Supplementary material for: Detailed Analysis of Sequence Changes Occurring during vlsE Antigenic Variation in the Mouse Model of Borrelia burgdorferi Infection
Source: PLoS Pathog. 2009 Feb 13;5(2):e1000293. doi: 10.1371/journal.ppat.1000293 (PMC2632889; doi:10.1371/journal.ppat.1000293)
Supplement: Table S2 — B. burgdorferi vlsE variant clones with sequences consistent with a single recombination event. (0.11 MB PDF) [file ppat.1000293.s002.pdf]

**Table S2.** *B. burgdorferi vlsE* variant clones with sequences consistent with a single recombination event.							
**Clone**	**Potential *vls* Cassette(s) Used (Longest Max. Recombination)**	**Nonambiguous (Only One Possible Cassette)**	**5' Extension for Max. Recombination (nt)**	**Minimum recombination (nt)**	**3' Extension for Max. Recombination (nt)**	**Maximum recombination (nt)**	**NCBI Accession Number**
D7M4B02	7	Yes	97	-6	8	99	EU484643
SD14M3B3 (same as D28M1B3b)	9	Yes	25	1	2	28	EU485400
D7M4J13	9	Yes	25	1	9	35	EU484817
D10M11S2	5	Yes	32	1	4	37	EU485035
D7M6H03	5	Yes	32	1	4	37	EU484726
D7M5J13	12	Yes	89	1	15	105	EU484821
SD10M3HX9	11	Yes	11	2	10	23	EU485646
D7M3J06	13	Yes	80	3	0	83	EU484777
SD14M2JX4	13	Yes	13	3	80	96	EU485799
D10M11B1	5	Yes	32	6	81	119	EU484853
SD14M4H3	9	Yes	77	7	8	92	EU485743
D7M1H03	7	Yes	6	8	76	90	EU484689
D7M1H04	7	Yes	6	8	76	90	EU484690
D10M9B11	13	Yes	76	8	8	92	EU484891
D7M4B03	9	Yes	86	8	31	125	EU484644
D7M6J10	9	Yes	86	8	31	125	EU484799
D10M11S5	4	Yes	13	10	25	48	EU485038
D10M9J5	4	Yes	13	10	25	48	EU485019
D7M6J05	14	Yes	0	10	76	86	EU484798
D7M2J05	10	Yes	2	11	6	19	EU484770
SD14M1HX5	3	Yes	12	11	76	99	EU485766
SD28M1S06	11	Yes	11	13	17	41	EU485973
D7M1B09	15	Yes	5	13	54	72	EU484622
1395F skin	8	Yes	69	13	17	99	EU485966
D10M9H4	13	Yes	0	14	7	21	EU484957
D10M9H6	13	Yes	1	14	7	22	EU484959
D7M2J12	5	Yes	76	14	7	97	EU484807
D10M10J3	7	Yes	7	15	6	28	EU484967
D7M3B12	12	Yes	0	16	9	25	EU484671
D10M11B4	9	Yes	77	16	7	100	EU484858
SD10M1EX5	8	Yes	21	16	79	116	EU485701
SD14M1BX3	10	Yes	1	17	86	104	EU485720
D7M1J08	10	Yes	21	19	86	126	EU484763
SD10M1EX11	10	Yes	21	19	86	126	EU485697
D10M8J9	12	Yes	83	20	7	110	EU485012
D28M3B04	4	Yes	25	21	9	55	EU485357
D10M7B6	6	Yes	39	22	63	124	EU484877
D10M11H4	6	Yes	61	22	83	166	EU484919
D7M6J14	6	Yes	8	23	61	92	EU484828
D7M4H03	9	Yes	40	38	2	80	EU484710
SD14M2H7	5	Yes	30	38	76	144	EU485740
D7M6J16	7	Yes	6	40	33	79	EU484830
SD28M4HX8	15	Yes	17	46	9	72	EU485941

Clone	Potential *vls* Cassette(s) Used (Longest Max. Recombination)	Nonambiguous (Only One Possible Cassette)	5' Extension for Max. Recombination (nt)	Minimum recombination (nt)	3' Extension for Max. Recombination (nt)	Maximum recombination (nt)	NCBI Accession Number
1379D skin	6	Yes	63	55	61	179	EU485850
D28M3B02	6	Yes	63	55	61	179	EU485355
SD28M3BX2	6	Yes	63	55	61	179	EU485874
SD10M4BX7	7	Yes	4	82	8	94	EU485627
SD28M3JX1	9	Yes	6	89	8	103	EU485956
D10M10B2	15	Yes	54	93	18	165	EU484845
D10M10J10	11	Yes	6	98	7	111	EU484962
D4M5S02	3	Yes	21	102	8	131	EU484594
D14M2B1	4	Yes	62	347	10	419	EU485089
D10M9J3	7,(8)	No	75	-3	9	81	EU485018
SD7M2HX3	3-7,(6),9,11-14,16	No	24	-3	63	84	EU485018
SD28M3BX7	3-7,9,11,(12),13,14,16	No	72	-3	16	85	EU485881
D14M5S9	(2),5,7,11,13	No	3	1	7	11	EU485247
SD10M3HX16	(2),5,7,11,13	No	3	1	8	12	EU485634
D7M5B05	10,12, (14)	No	7	1	7	15	EU484654
D7M2B08	2-5,7,9,11,12,13 (14)	No	11	1	7	19	EU484631
D7M4J11	2-5,7,9,11,12,13 (14)	No	11	1	7	19	EU484815
D10M7S8	5,7,(8),10,16	No	16	1	13	30	EU485059
D7M3J07	5,7,(8),10,16	No	16	1	13	30	EU484778
SD14M3HX5	5,7,(9),10	No	6	1	31	38	EU485764
SD28M4HX7	2,(4),11,13	No	41	1	4	46	EU485940
SD14M3J11	3,4,6,11,12,13,(14),15	No	15	1	48	64	EU485795
D7M3J17	9,12,(13),15	No	46	1	20	67	EU484814
D10M7J6	3-5,8,9,11,13,(15)	No	17	1	54	72	EU484999
SD14M3J6	3,4,5,8,9,11,13,(15)	No	17	1	54	72	EU485780
D14M5S7	(4),5,9,10,11,12,14,15,16	No	61	1	22	84	EU485245
D10M10B11	3,5,(6),7,9,11,14	No	8	1	79	88	EU484842
SD14M4H2	(3),11,13	No	75	1	12	88	EU485742
D10M7S6	(10),14	No	6	1	83	90	EU485057
D7M4H07	(10), 14	No	6	1	83	90	EU484714
D10M11H12	3,5,(6),7,9,11,14	No	8	1	83	92	EU484916
SD14M1HX8	4,5,(14)	No	7	1	88	96	EU485758
D10M10J1	4-7,9,(10),11,14-16	No	20	1	77	98	EU484961
SD14M3HX3	4,5,6,7,9,(10),11, 14,16	No	20	1	77	98	EU485763
D10M7B2	2-3,(4),5-9,13,15,16	No	80	1	18	99	EU484875
D10M8J10	2,3,(4),5-9,13,15,16	No	80	1	18	99	EU485004
D7M1J01	2-3,(4),5-9,13,15,16	No	80	1	18	99	EU484759

Clone	Potential *vls* Cassette(s) Used (Longest Max. Recombination)	Nonambiguous (Only One Possible Cassette)	5' Extension for Max. Recombination (nt)	Minimum recombination (nt)	3' Extension for Max. Recombination (nt)	Maximum recombination (nt)	NCBI Accession Number
SD14M2J12	2-9,13,15,16(4)	No	80	1	18	99	EU485776
D10M10J23	2,3,6,8,11,(12),13,15,16	No	89	1	15	105	EU484966
D7M1B03	2-3,6,8,11,(12), 13,15,16	No	89	1	15	105	EU484618
D7M1J11	2-3,6,8,11,(12), 13,15,16	No	89	1	15	105	EU484800
D10M11S3	(7),9	No	10	2	25	37	EU485036
D10M11J7	7,9,10,11,(12)	No	13	2	33	48	EU484979
D10M12S3	7,9,10,11,(12)	No	13	2	33	48	EU485047
SD28M1HX1	7,9,10,11,(12)	No	13	2	33	48	EU485906
D7M2J08	3,8,(11),13-16	No	17	2	32	51	EU484773
SD10M4BX5	6,(8),12	No	11	2	73	86	EU485625
SD14M1BX7	6,(8),12	No	11	2	73	86	EU485723
SD7M2JX10	2,(4),12	No	0	2	88	90	EU485589
D10M7J4	(8),13	No	10	2	85	97	EU484998
D10M9H11	(6),9	No	63	2	63	128	EU484951
D10M10S5	3,4,(6),13-15	No	79	3	18	100	EU485027
D10M7H4	3,4,(6),13,14,15	No	79	3	18	100	EU484939
SD10M2BX10	3,4,(6),13,14,15	No	79	3	18	100	EU485603
SD14M2B2	3,4,(6),13,14,15	No	79	3	18	100	EU485710
SD28M4BX20	3,4,(6),13,14,15	No	79	3	18	100	EU485893
D7M5J07	3,4,10,11,(12)	No	10	3	88	101	EU484794
SD7M2JX1	3,4,10,(12)	No	10	3	88	101	EU485575
D10M12B1	2,(4),5,9,11,12	No	12	3	88	103	EU484863
D10M12B7	2,(4),5,9,11,12	No	12	3	88	103	EU484871
SD10M3HX10	2,6,8,(16)	No	8	4	16	28	EU485630
D14M2S9	3,4,5,(6),8,12,14	No	16	4	86	106	EU485236
SD10M4HX2	9,(12)	No	7	5	8	20	EU485648
D7M6J12	(9),11	No	31	5	4	40	EU484826
D10M8J3	(3),4,5,13	No	13	5	30	48	EU485006
D10M9B22	(3),4,5,13	No	13	5	30	48	EU484894
SD14M2JX1	(3),4,5,13	No	13	5	30	48	EU485788
D14M1J2	4,5	No	2	5	61	68	EU485171
SD28M2JX7	5,9,(11)	No	6	5	76	87	EU485954
SD14M1BX10	13,(14)	No	76	5	11	92	EU485717
SD14M4E2	13,(14)	No	76	5	11	92	EU485821
D7M1B12	(3),8,10,13,16	No	21	6	7	34	EU484666
SD7M1JX1	2-5,(7),810-14,16	No	33	6	7	46	EU485574
D10M12H2	5,9,(11)	No	6	6	75	87	EU484927
D10M11S8	3,4,(6),11,13,15	No	3	7	61	71	EU485041
D14M1J4	4,5	No	16	8	61	85	EU485172
D10M7J8	2,3,6,8,13,(15),16	No	80	10	9	99	EU485001
SD28M4HX6	2,3,6,8,13,(15),16	No	80	10	9	99	EU485939
SD10M3HX15	(7),10	No	19	12	33	64	EU485633

Clone	Potential *vls* Cassette(s) Used (Longest Max. Recombination)	Nonambiguous (Only One Silent Cassette)	5' Extension for Max. Recombination (nt)	Minimum recombination (nt)	3' Extension for Max. Recombination (nt)	Maximum recombination (nt)	NCBI Accession Number
D7M6B02	(2),7	No	83	14	7	104	EU484658
SD28M1HX2	(7),10	No	19	12	33	64	EU485912
D10M10S11	(2),10	No	34	18	9	61	EU485023
D7M1B10	3,4,(13)	No	5	19	30	54	EU484623
1395A skin	3,11,(13)	No	0	20	10	30	EU485962

a All silent cassettes that could have served as template for the recombination event are listed. If only one silent cassette was identical with the minimum recombination region, the recombination event was considered unambiguous. In ambiguous cases, all silent cassettes listed could have served as the template for the recombination event (i.e. have identical minimum recombination regions). The silent cassette with the longest possible maximum recombination (in parentheses) was selected for calculation of maximum recombination values shown. All values represent the minimum and maximum recombination event lengths in the variant sequence. Negative numbers in the minimum recombination column represent deletions in the variant sequence relative to the parent sequence.
