# Supplementary material for: Detailed Analysis of Sequence Changes Occurring during vlsE Antigenic Variation in the Mouse Model of Borrelia burgdorferi Infection
Source: PLoS Pathog. 2009 Feb 13;5(2):e1000293. doi: 10.1371/journal.ppat.1000293 (PMC2632889; doi:10.1371/journal.ppat.1000293)
Supplement: Table S3 — Comparison of the base changes occurring in ‘templated’ vs. ‘template-independent’ single nucleotide changes in vlsE variants. (0.08 MB PDF) [file ppat.1000293.s003.pdf]

Table S3. Comparison of the base changes occurring in 'templated' vs. 'template-independent' single nucleotide changes in *vlsE* variants. [a]

**'Templated' single nucleotide changes (N = 33)**					
		Nucleotide change (proportion)			
Initial nucleotide		A	T	G	C
	A		0 (0)	10 (0.30)	3 (0.09)
	T	0 (0)		2 (0.06)	0 (0)
	G	4 (0.12)	2 (0.06)		0 (0)
	C	11 (0.33)	0 (0)	1 (0.03)	
**'Template-independent' single nucleotide changes (N = 76)**					
		Nucleotide change (proportion)			
Initial nucleotide		A	T	G	C
	A		2 (0.03)	17 (0.22)	8 (0.11)
	T	3 (0.04)		7 (0.09)	3 (0.04)
	G	10 (0.13)	5 (0.07)		6 (0.08)
	C	0 (0)	12 (0.16)	3 (0.04)	

a For the 'templated' group, well-defined single nucleotide changes that were clearly separate from other sequence variations were used. Shaded boxes indicate those changes that differed substantially between the two types.
