# Supplementary material for: Detailed Analysis of Sequence Changes Occurring during vlsE Antigenic Variation in the Mouse Model of Borrelia burgdorferi Infection
Source: PLoS Pathog. 2009 Feb 13;5(2):e1000293. doi: 10.1371/journal.ppat.1000293 (PMC2632889; doi:10.1371/journal.ppat.1000293)
Supplement: Figure S2 — Locations of template-independent sequence variation. The X axis numbers represent the codon number of each amino acid in vlsE as presented in Figure 3 of [22]. The dark blue bars represent the number of variants recovered at each codon. The black line represents a 3 point moving average. In the diagram below the graph, the light areas indicate the locations of the invariable regions of vlsE while the dark areas indicate the positions of the variable regions of vlsE. (0.07 MB PDF) [file ppat.1000293.s005.pdf]

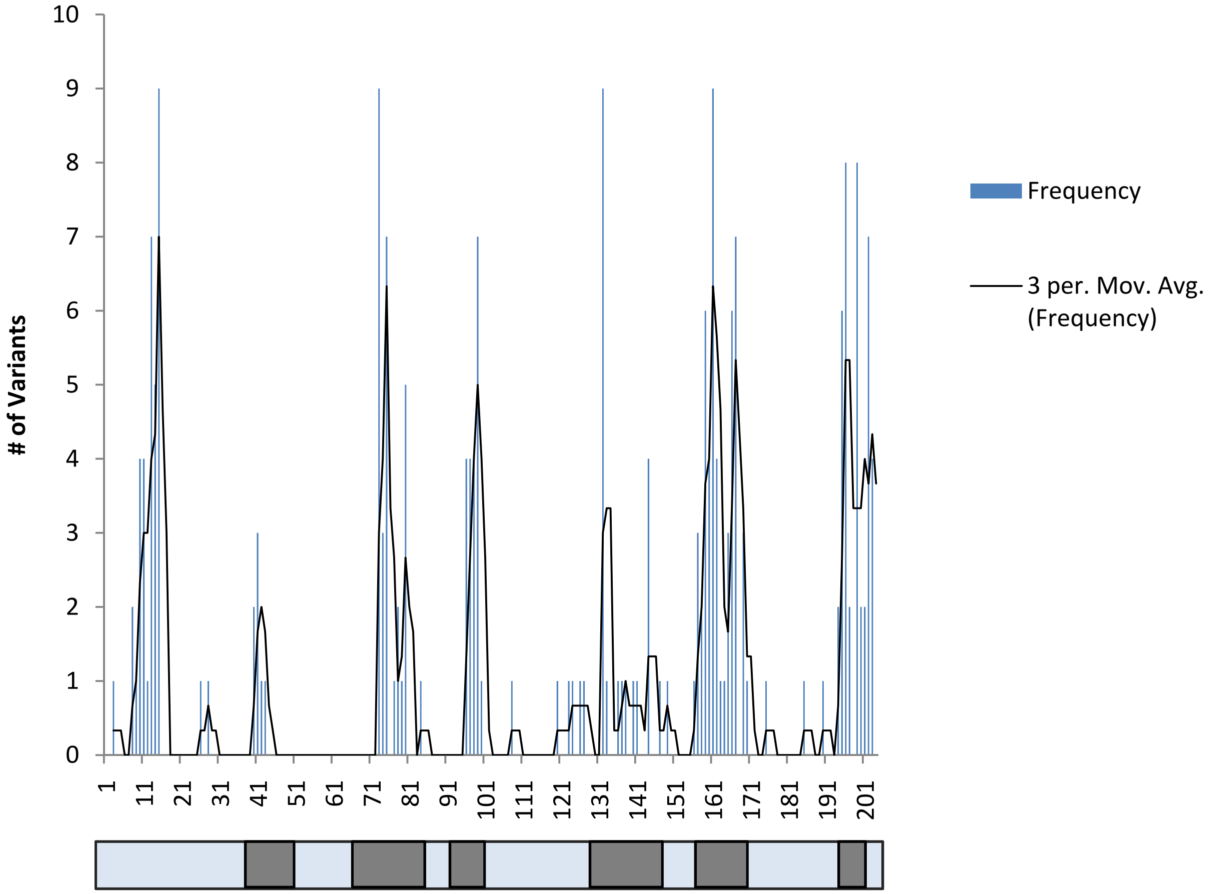

Fig. S2. Locations of template-independent sequence variation. The X-axis numbers represent the codon number of each amino acid in *vlsE* as presented in figure 3 of [22]. The dark blue bars represent the number of variants recovered at each codon. The black line represents a 3 point moving average. In the diagram below the graph, the light areas indicate the locations of the invariable regions of *vlsE* while the dark areas indicate the positions of the variable regions of *vlsE*.
